# Effects of Sodium Alginate on the Quality Attributes of Wet Instant Starch Noodle Cakes: From a Multi-Scale Perspective

**DOI:** 10.3390/foods15193490

**Published:** 2026-09-29

**Authors:** Huanhuan Zhang, Ling Zhang, Sheng Li, Shixiong Yang, Yexing Liang, Xiaofeng Zeng, Feihu Gao

**Affiliations:** 1 Institute of Agricultural Products Processing, Chongqing Academy of Agricultural Sciences, Chongqing 401329, China; jdteazl@163.com (L.Z.); yangsx1019@163.com (S.Y.); 18983692739@163.com (Y.L.); xndxzxf@126.com (X.Z.); 2 Chongqing Academy of Chinese Materia Medica, Chongqing University of Chinese Medicine, Chongqing 400065, China; lsheng1990@126.com

**Keywords:** wet instant starch noodle cakes (WISNC), sodium alginate, rheological properties, cooking performance, multi-scale structure

## Abstract

This study aimed to investigate the potential of sodium alginate (SA) as a substitute for potassium alum (PA) to improve the quality of wet instant starch noodle cakes (WISNC). The effects of SA on the rheological properties of the starch dough, as well as the rehydration time, cooking performance, textural properties, multi-scale structure, and water distribution of the resulting WISNC, were systematically evaluated. The results demonstrated that the effects of SA on the quality attributes of WISNC were concentration-dependent, with 0.2% SA exhibiting the most pronounced improvement by effectively reducing cooking loss and shortening rehydration time while maintaining desirable textural properties. Rheological analysis showed that the addition of 0.2% SA effectively enhanced starch dough strength. Multi-scale structural analyses based on FTIR, Raman, XRD, DSC, and SEM revealed that an appropriate level of SA addition improved WISNC quality by promoting starch chain alignment and structural ordering, enhancing hierarchical structural organization, facilitating the formation of a more homogeneous starch–SA gel network, and mitigating freeze-induced structural damage caused by ice crystal formation. These findings provide mechanistic insights into starch–SA interactions and highlight the potential of SA as a functional alternative to PA for improving the quality attributes of WISNC.

## 1. Introduction

Hot and sour starch noodles (HSSNs), also known as Suanlafen, are a popular starch-based food in China, prepared primarily from sweet potato starch and characterized by their distinctive sour and spicy flavor. Driven by modern lifestyles and the consequent increasing consumer demand for convenient foods [1], instant HSSNs have gained considerable popularity. The starch noodle cakes serve as the core structural component of instant HSSNs, and their rehydration time and cooking properties contribute to the quality attributes of the final product. To extend their shelf life, conventional starch noodle cakes are typically processed through dehydration to achieve a dried state. However, dehydration not only requires high energy consumption [2] but may also induce shrinkage of the starch gel network and deterioration of sensory quality, while prolonging the rehydration time before serving [3,4]. Therefore, wet instant starch noodle cakes (WISNC), which avoid the conventional drying process and maintain desirable flexibility and elasticity, have attracted increasing research interest in starch-based convenience foods.

WISNC are manufactured via the dropping method [5], which involves starch dough preparation, dropping, cooking, cooling, molding, aging, demolding, freezing, thawing, and vacuum packaging. Among these processing steps, freezing is indispensable for noodle separation [6], but remains a critical determinant of product quality, as uncontrolled ice crystal formation and growth during freezing can damage the starch gel network, thereby increasing broken strip rate and cooking loss. To improve the quality of WISNC, alum (KAl(SO_4_)_2_·12H_2_O) is typically added as a modifier [7]. However, excessive aluminum exposure may promote the development of osteoporosis and neurodegenerative conditions [8,9], posing a potential threat to human health. Therefore, exploring safe and healthy alternative additives to improve the quality of WISNC holds significant practical importance and application value.

Previous studies have confirmed that the addition of hydrocolloids is an effective approach to improving the texture and cooking quality of gluten-free noodles, e.g., starch noodles and rice noodles [10,11,12], with the effectiveness depending on the type and addition level of the hydrocolloids. Sodium alginate (SA) is a polysaccharide-based hydrophilic colloid extracted from the structural matrix and cell walls of brown algae [13]. This linear polymer consists of two epimeric monomers, α-L-guluronic acid (G) and β-D-mannuronic acid (M), which are connected through 1,4-glycosidic linkages [14]. The M and G residues are connected in either homogeneous or mixed patterns, forming polymannuronate (poly M), polyguluronate (poly G), and alternating copolymer (poly MG) blocks [15]. Compared with many other common hydrocolloids, SA is cost-effective and is widely applied in starch-based systems due to its multifunctionality, particularly its superior gelling and thickening abilities [16] as well as relatively good freeze–thaw stability. For example, Rivero-Ramos et al. [17] prepared a specialized food matrix suitable for patients with dysphagia by adding SA to common rice starch. Feng et al. [18] added SA to wet sweet potato starch noodles and found that the tensile strength, elongation, and cooking break time of the noodles were significantly improved. Feng et al. [19] further found that the SA-chitosan complex system, as a composite ionic polysaccharide, imparted the strongest tensile properties to wet sweet potato starch noodles. Although SA has shown potential in starch noodle systems, its effects on the quality improvement of WISNC, which undergo freezing, remain rarely reported. Moreover, the mechanisms by which SA regulates the structural characteristics responsible for WISNC quality remain poorly understood.

Therefore, the objectives of this study are twofold: (1) to evaluate the effects of SA on the rehydration time, cooking quality, and textural properties of WISNC; (2) to elucidate the mechanisms underlying the effects of structural changes on the quality attributes of WISNC from the perspectives of starch dough rheological properties, starch multiscale structure, and water distribution. These findings are anticipated to offer a theoretical foundation and practical guidance for replacing alum with SA and improving the quality attributes of WISNC.

## 2. Materials and Methods

### 2.1. Materials and Reagents

Sweet potato starch (moisture content: 11.14%; dry basis composition: 94.54% starch, 18.10% amylose (*w*/*w* of total starch), 0.12% crude protein, and 0.35% ash) was obtained from Chongqing Hanping Food Co., Ltd. (Chongqing, China). SA (food grade) was obtained from Qingdao Mingyue Seaweed Group Co., Ltd. (Qingdao, China), with a viscosity of 150 mPa·s (1%, 20 °C) and an assay of 99%. Potassium alum (PA) was obtained from Wenzhou Alum Mine Development Group Co., Ltd. (Wenzhou, China). Spectral-grade KBr was acquired from Sinopharm Group Co., Ltd. (Beijing, China). The Amylose/Amylopectin Assay Kit was purchased from Megazyme (Bray, Ireland). Reagents not specifically listed were of analytical grade.

### 2.2. Preparation of WISNC

The preparation of WISNC was designed according to the industrial production scheme [20], with laboratory-scale modifications. The detailed process flow is illustrated in Figure S1. Briefly, 30 g of sweet potato starch (SPS) was mixed with 30 g of distilled water to form a slurry. After this, 450 g of boiling water was added, and the mixture was heated with continuous stirring until a transparent viscous paste formed. Then 150 mL of distilled water was added, followed by the remaining 1125 g of starch after thorough mixing. After preliminary mixing, the mixture was transferred to a dough mixer (Bear, Foshan, China). During stirring, SA solutions (0.1%, 0.2%, 0.3%, and 0.4% of total starch, each in 150 mL distilled water) and a PA solution (0.3% of total starch [18] in 150 mL distilled water) were added. A sample without PA or SA, with the same amount of water added (150 mL), served as the Control. Additional water was added as needed, with 20, 45, 51, 75, 82, and 41 g added to the Control, 0.1% SA, 0.2% SA, 0.3% SA, 0.4% SA, and 0.3% PA groups, respectively. Mixing was continued until a uniform, smooth starch dough that flowed continuously when lifted was obtained. Then, the mixed starch dough was pressed through an aluminum ladle with five holes (20 mm in diameter) at a height of approximately 30 cm into boiling water. After floating, the noodles were cooked for an additional 15 s, then cooled in room-temperature water (with water changes). After draining, 100 g portions were placed into 300 mL perforated plastic bowls. The noodle cakes were sequentially aged at ambient temperature for 2 h, at 4 °C for 4 h, and then frozen at −7 °C for 16 h. Subsequently, a portion of the frozen noodle cakes were freeze-dried, pulverized, and passed through a 200-mesh screen for multi-scale structural characterization. The remaining frozen noodle cakes were thawed at room temperature for 2 h, air-dried with a cooling fan to a moisture content of approximately 56 g/100 g across all groups, and then vacuum-packaged to obtain the final WISNC (the moisture content of the Control, 0.1% SA, 0.2% SA, 0.3% SA, 0.4% SA, and 0.3% PA WISNC samples was 55.94 ± 1.24, 56.76 ± 0.64, 56.13 ± 1.16, 57.04 ± 0.26, 56.29 ± 0.32, and 55.92 ± 0.70 g/100 g, respectively, with no significant differences observed among the groups). Following this, the water distribution, rehydration time, sensory evaluation, cooking quality, and textural properties of WISNC were determined.

### 2.3. Rheological Behavior of Starch Dough for WISNC Production

The rheological behavior of the starch dough was determined following the protocol of Zhang et al. [21] with minor adjustments. An MCR92 rheometer (Anton Paar, Graz, Austria) fitted with a 25 mm smooth parallel plate was used, with the plate separation set at 1 mm. Following sample placement, excess dough around the plate was removed using a matching scraper. Silicone oil was then applied around the exposed sample surface to minimize moisture loss, and the samples were allowed to rest for 1 min before the measurement.

#### 2.3.1. Oscillatory Amplitude Sweep Test

The linear viscoelastic region (LVER) of starch dough was characterized via an amplitude sweep test at 25 °C, using a strain amplitude range of 0.01–10% and an angular frequency of 10 rad/s. The LVER was calculated using the “LVE range” method in the RheoCompass software (version 1.36.2226-Release), with strain and storage modulus G′ taken as the calculation variables and a boundary deviation ratio of 3% being set.

#### 2.3.2. Oscillatory Frequency Sweep Test

Frequency sweep tests were conducted at 25 °C across an angular frequency range of 0.1–100 rad/s, at a constant strain (based on the proposed strain value determined from the amplitude sweep test) within LVER. Variations in *G*′ and *G*″ as a function of frequency were recorded. The power-law model given in Equation (1) [22] was employed to fit *G*′ to quantitatively characterize the frequency dependence of *G*′ and dough strength.

log*G*′ = *m*log*ω* + *n* (1)

where *G*′ refers to the storage modulus, *ω* signifies the angular frequency, log denotes log_10_, and *m* and *n* are constants obtained by fitting each replicate independently. The resulting *m* and *n* values were subsequently subjected to statistical analysis.

### 2.4. Rehydration Time of WISNC

The determination of rehydration time was conducted according to GB/T 23587–2024 [23], with some modifications. Four WISNC samples were placed in separate lidded noodle bowls of identical specifications, and boiling water was simultaneously poured into each bowl until the noodle cakes were just submerged. The lids were immediately covered, and a stopwatch was started. Starting from one minute before the expected rehydration time, one bowl was opened every 15 s. For each opened bowl, a piece of noodle was taken out using chopsticks and was laid flat on a white comparison plate. When the noodle in a bowl was observed to appear uniformly translucent, showed no white hard core upon pressing, and had no raw taste upon tasting, the rehydration time corresponding to that bowl was recorded. This recorded time was taken as the rehydration time.

### 2.5. Cooking Loss of WISNC

The determination of cooking loss was performed following Li et al. [24] with some modifications. The WISNC sample (*M*_0_) was placed in 900 mL of boiling water and boiled for 1 min 40 s. After boiling, the starch noodles were filtered out, and the cooking liquid was gathered in a 1000 mL volumetric flask, which was then adjusted to the mark using distilled water. Subsequently, an aliquot of 100 mL of the cooking liquid was transferred into a weighing bottle (*M*_1_) that had been cleaned and dried to a stable mass. The cooking liquid was evaporated at 105 °C until a constant weight was achieved, after which the mass was recorded as *M*_2_. Cooking loss was quantified following Equation (2) [14].

(2) $$\mathrm{Cooking}\;\mathrm{loss}\;\left(\%\right)=\frac{M_{2}-M_{1}}{M_{0}\times (1-\mathrm{moisture}\;\mathrm{of}\;\mathrm{WISNC})}\times 10\times 100$$

### 2.6. Texture Properties of WISNC

The cooked WISNCs (Section 2.5) were immediately cooled with running water, and the surface water was blotted with kitchen paper towels. Subsequently, texture profile analysis (TPA) was performed with a CT3 texture analyzer (Brookfield, Middleborough, MA, USA). The experimental method was based on the method reported by Xiang et al. [5] with slight modifications. Briefly, the samples were trimmed to a uniform length of 8 cm. For every sample, three strands of starch noodles were positioned side by side on the fixture panel, with a 1 cm interval separating adjacent noodles. Using a TA4/1000 probe, the samples were subjected to compression at 1 mm/s until their height was reduced to 60% of the original, with a trigger force of 4.0 g. The texture parameters, namely hardness and cohesiveness, were systematically assessed in triplicate for all samples.

### 2.7. Fourier Transform Infrared Spectroscopy (FTIR)

FTIR spectra were acquired on a Nicolet iS20 spectrometer (Thermo Fisher Scientific, Waltham, MA, USA) from 400 to 4000 cm^−1^ at 4 cm^−1^ resolution with 32 scans [25]. Baseline correction and subsequent spectral deconvolution over 1200–800 cm^−1^ were carried out using OMNIC 9.0 (Thermo Fisher Scientific, Waltham, MA, USA).

### 2.8. Laser Confocal Micro Raman Spectroscopy

Raman spectra were acquired using a LabRAM HR Evolution Raman spectrometer (HORIBA Scientific, Villeneuve-d’Ascq, France), following the method reported by Yang et al. [26] with minor adjustments. A 532 nm laser served as the excitation source, and spectra spanning 100–3500 cm^−1^ were recorded with 10 scans, each lasting 20 s.

### 2.9. X-Ray Diffraction (XRD)

The crystal structure of the samples was characterized by an XRD-7000 X-ray diffractometer (Shimadzu, Kyoto, Japan) equipped with a Cu Kα radiation source. The measurements were performed with a 2°/min scan rate, covering the 2θ interval from 5° to 40° [27]. Relative crystallinity was quantified using Jade 6.0 (Materials Data, Inc., Livermore, CA, USA).

### 2.10. Differential Scanning Calorimeter (DSC)

Thermal analysis was conducted using a DSC 300 (NETZSCH, Selb, Germany) as per Gao et al. [28] with slight alterations. Samples (2.8–3.0 mg) were combined with 6.0 μL of deionized water, enclosed within aluminum pans, and then kept at 4 °C for 24 h prior to measurement. The temperature was ramped from 30 to 105 °C at 10 °C/min. Heat flow curves were analyzed via NETZSCH Assistant 9 to extract the onset (*T*o), peak (*T*_p_), and conclusion (*T*_c_) temperatures, along with the enthalpy change (Δ*H*) of the phase transition.

### 2.11. Low-Field Nuclear Magnetic Resonance (LF-NMR)

The water distribution in WISNC products was determined by an NMI20-060H-I LF-NMR analyzer (Niumag Corporation, Suzhou, China). Samples of 6–8 g were taken from round WISNC and placed in a 20 mm glass tube. T_2_ relaxation measurements were carried out via a Carr-Purcell-Meiboom-Gill (CPMG) pulse sequence with the following settings: echo time = 0.1 ms, echoes = 5000, gain = 1, gain level = 1, waiting time = 2500 ms, scans = 4, sampling bandwidth = 200 kHz. Relaxation data were inverted using a multi-component model, BRD method, and a smoothing factor of 0.1.

### 2.12. Scanning Electron Microscope (SEM)

Cross-sections of WISNC samples were imaged with a GeminiSEM 360 (Carl Zeiss AG, Oberkochen, Germany) following Xiang et al. [5] with slight modifications. Prior to imaging, the samples were sputter-coated with platinum using a JY-4108H ion sputter coater (Guangzhou Jingying Scientific Instrument Co., Ltd., Guangzhou, China). Micrographs were captured at magnifications of 20× and 100×.

### 2.13. Determination of Aluminum Content

The aluminum content was determined by inductively coupled plasma mass spectrometry (SUPEC 7000, EXPEC, Hangzhou, China) according to GB 5009.182–2017 [29].

### 2.14. Statistical Analysis

All tests were performed in triplicate, and the data are expressed as mean ± standard deviation (SD). Statistical comparisons were conducted in SPSS 27.0 (IBM SPSS Statistics, Chicago, IL, USA) using one-way ANOVA followed by Duncan’s post hoc test. Statistical significance was defined as *p* < 0.05. All data were first tested for normality and homogeneity of variance prior to ANOVA. Figures were prepared in Origin 2024 (OriginLab, Northampton, MA, USA) and Adobe Illustrator 2025 (Adobe, San Jose, CA, USA).

## 3. Results and Discussion

### 3.1. Rheological Properties of Starch Doughs

#### 3.1.1. Oscillatory Strain Sweep

**Figure 1:**
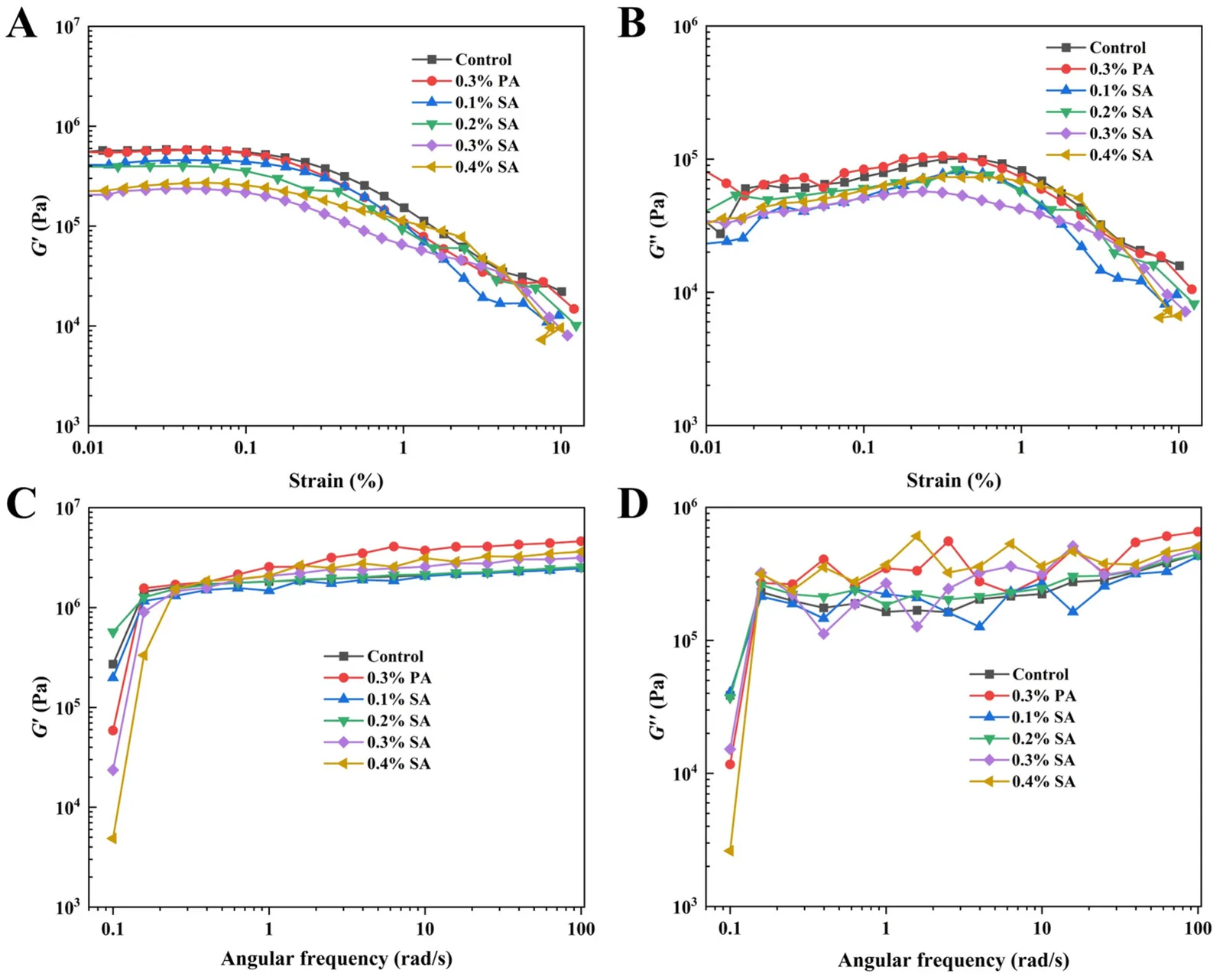
Oscillatory strain sweep and frequency sweep data for starch doughs supplemented with PA and various concentrations of SA. (**A**) Storage modulus (*G*′) versus strain; (**B**) loss modulus (*G*″) versus strain; (**C**) *G*′ versus angular frequency; (**D**) *G*″ versus angular frequency. Control: starch dough without additives; 0.3% PA: starch dough containing 0.3% potassium alum (*w*/*w*); 0.1–0.4% SA: starch dough containing the corresponding concentrations of sodium alginate (*w*/*w*).

The influence of PA and different SA addition levels on the rheological properties of starch dough was examined based on viscoelastic behavior. Amplitude sweep curves of each starch dough sample were presented in Figure 1A,B. The LVER of each sample was determined by analyzing the plateau region of *G*′, with the results summarized in Table 1. The upper limits of LVER for all starch dough groups ranged from 0.018% to 0.068%, slightly broader than the 0.031–0.045% reported by Feng et al. [18], which may be attributed to differences in dough formulations or measurement parameters, such as the plate gap. Compared to the LVER of wheat flour dough (0.10–0.15%) [30,31], starch dough used in WISNC production exhibits a lower upper limit. This is attributed to the firm and stable gluten network that characterizes wheat flour dough, whereas starch dough, prepared by mixing a small amount of pre-gelatinized starch paste with native starch and water, is typically composed of a fragile and friable polysaccharide network [21].

As shown in Table 1, LVER values did not differ significantly between the control and the 0.1%, 0.3%, or 0.4% SA-supplemented groups (*p* > 0.05). Conversely, the addition of PA or 0.2% SA significantly elevated LVER values (*p* < 0.05), suggesting that these additives improve the structural stability of starch dough. To interpret this result, the starch dough can be regarded as a biphasic system, in which starch granules are distributed within a continuous binder matrix, and the binder forms a thin, low-shear-strength film between the granules [32]. Both Al(OH)_3_ generated by PA hydrolysis and an appropriate amount of SA enhanced the strength of this film, thereby rendering the starch dough structure more stable.

#### 3.1.2. Oscillatory Frequency Sweep

Figure 1C,D depict the frequency dependence of *G*′ and *G*″ for all starch doughs. Within the 0.1–100 rad/s interval, all samples exhibited *G*′ values exceeding *G*″, and both moduli exhibited a sharp increase at low frequencies followed by a more gradual rise at higher frequencies, indicating solid-like elastic behavior. Similar findings were documented by Feng et al. [18] upon addition of alum and various polysaccharides to sweet potato starch dough. The frequency dependence of *G*′ was described by the power-law relationship log *G*′ = *m* log *ω* + *n*. The exponent m reflects the frequency sensitivity of the network structure: A higher m value indicates that the system is more sensitive to frequency variations, reflecting weaker physical interactions within the network structure and relatively lower structural stability [18]. The coefficient n reflects the overall magnitude of *G*′ and is commonly used as an indicator of dough strength, with higher values corresponding to a stronger network structure [19]. *R*^2^ was used to evaluate the goodness of fit.

During fitting, the initial data point at 0.1 rad/s was excluded due to low-frequency instability, and the refitted parameters are shown in Table 1. The *m* values of starch doughs with different additives ranged from 0.085 to 0.126. This is in agreement with Feng et al. [19], who investigated starch dough systems incorporating ionic polysaccharides and egg white protein complexes, indicating that starch dough systems are primarily constructed through physically interacting network structures, which are relatively weak in overall stability and exhibit a certain degree of frequency dependence. Compared with the control, PA increased both *m* (0.116) and *n* (6.325), suggesting an overall increase in dough strength and a strengthened network structure, albeit with increased frequency dependence. At an SA level of 0.2%, m exhibited no significant change relative to the control, whereas n increased to 6.282, indicating an enhancement in network strength without altering its frequency dependence. In contrast, further increases in SA increased *m* and decreased *n*, indicating weakened network integrity and enhanced viscoelastic sensitivity to frequency, possibly due to molecular crowding, phase heterogeneity, and reduced effective starch–starch interactions [33] at high SA levels.

### 3.2. Rehydration Time and Cooking Loss Analysis of WISNC

**Figure 2:**
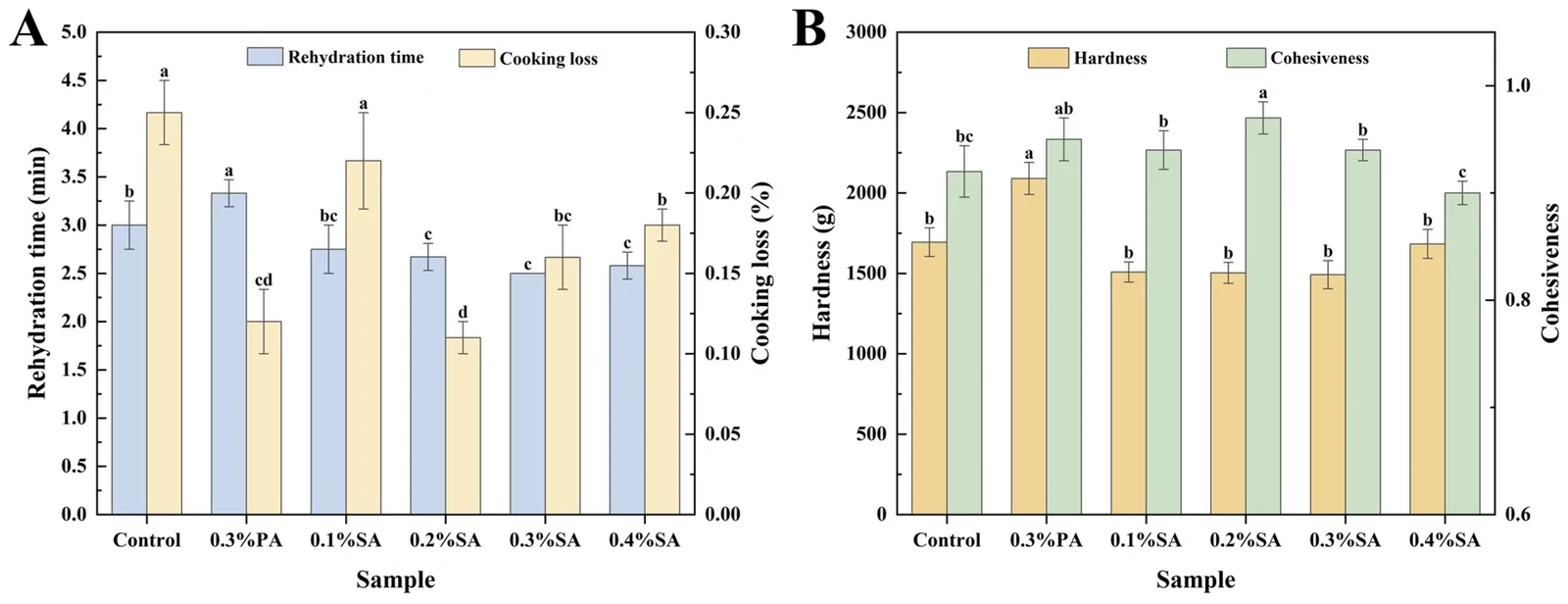
Rehydration time and cooking loss (**A**) and textural properties (**B**) of wet instant starch noodle cakes (WISNC). Different lowercase letters above bars of the same color indicate significant differences (*p* < 0.05). Control: WISNC without additives; 0.3% PA: WISNC containing 0.3% potassium alum (*w*/*w*); 0.1–0.4% SA: WISNC containing 0.1–0.4% sodium alginate (*w*/*w*).

An ideal instant starch noodle product should possess a short rehydration time and low cooking loss [3]. Figure 2A shows the effects of PA and varying SA concentrations on WISNC rehydration time and cooking loss (CL). PA supplementation markedly extended the rehydration time of WISNC compared to the control group. This is primarily ascribed to the creation of a tight gel network, resulting from the adsorption of starch molecular chains by aluminum hydroxide colloids generated through the hydrolysis of Al^3+^ [34,35], which impedes water penetration. As the SA content increased, the rehydration time initially decreased and then plateaued. This trend concurs with the findings of Xu et al. [36], who studied the effect of SA on the optimal cooking time of extruded whole buckwheat noodles. The observed behavior is ascribed to the abundant and strongly hydrophilic hydroxyl groups in SA molecules, which facilitate rapid water infiltration into the noodle interior, thereby shortening the rehydration time.

The CL of WISNC decreased from 0.25% to 0.12% after PA addition. This decrease is mainly attributed to the dense network structure formed by PA and starch molecules, which inhibits excessive swelling and disruption of starch granules, thereby reducing the leaching of solid matter. Compared with the control group, the addition of SA at levels of 0.2%, 0.3%, and 0.4% significantly reduced the CL, which is consistent with the report by Srikaeo et al. [37] that the addition of carboxymethyl cellulose hydrocolloid significantly reduced the CL of dried natural fermented rice noodles. Hong et al. [16] also found that SA supplementation at various levels significantly decreased the CL of highland barley-fortified wheat noodles. Furthermore, with increasing SA addition, the CL of WISNC first decreased and then increased, with the lowest value (0.11%) observed at the 0.2% addition level, which corresponded to 44% of that of the control group (0.25%), indicating an optimal addition level for SA. This may be attributed to the formation of stronger intermolecular associations within the starch–SA matrix at an appropriate SA addition level, which could enhance the structural integrity and compactness of the starch network, consequently reducing the release of starch components during cooking. However, at excessive SA concentrations, the increased polymer-chain density may have altered the organization of the starch–SA network through enhanced physical interactions, potentially resulting in greater structural heterogeneity and a concomitant increase in CL.

### 3.3. Texture Behavior Analysis of WISNC

The TPA focused on the hardness and cohesiveness of WISNC samples with different additive groups, and the results are presented in Figure 2B. In terms of hardness, the PA-added group exhibited a significantly higher value (2090.33 g) than both the control group (1694.00 g) and the SA-added groups (1491.80–1683.00 g), while supplementation with 0.1–0.4% SA exerted no significant effect relative to the control. Chen et al. [38] also reported that the addition of PA significantly increased the hardness of potato starch gel. Regarding cohesiveness, the 0.2% SA group (0.97) and the PA group (0.95) exhibited the highest values, whereas the 0.4% SA group (0.90) and the control group (0.92) showed the lowest. Notably, the addition of 0.2% SA increased cohesiveness without significantly altering hardness, suggesting that an appropriate level of SA may improve the structural integrity of WISNC. However, further increasing the SA concentration reduced cohesiveness, indicating that excessive SA addition may adversely affect the structural integrity of WISNC.

Taken together, the results of rehydration time, cooking quality, and textural properties indicated that SA had a concentration-dependent effect on WISNC quality. To elucidate the underlying structure–function relationships, a systematic investigation of starch multiscale structural evolution, together with water distribution, was therefore carried out.

### 3.4. FTIR Analysis

**Figure 3:**
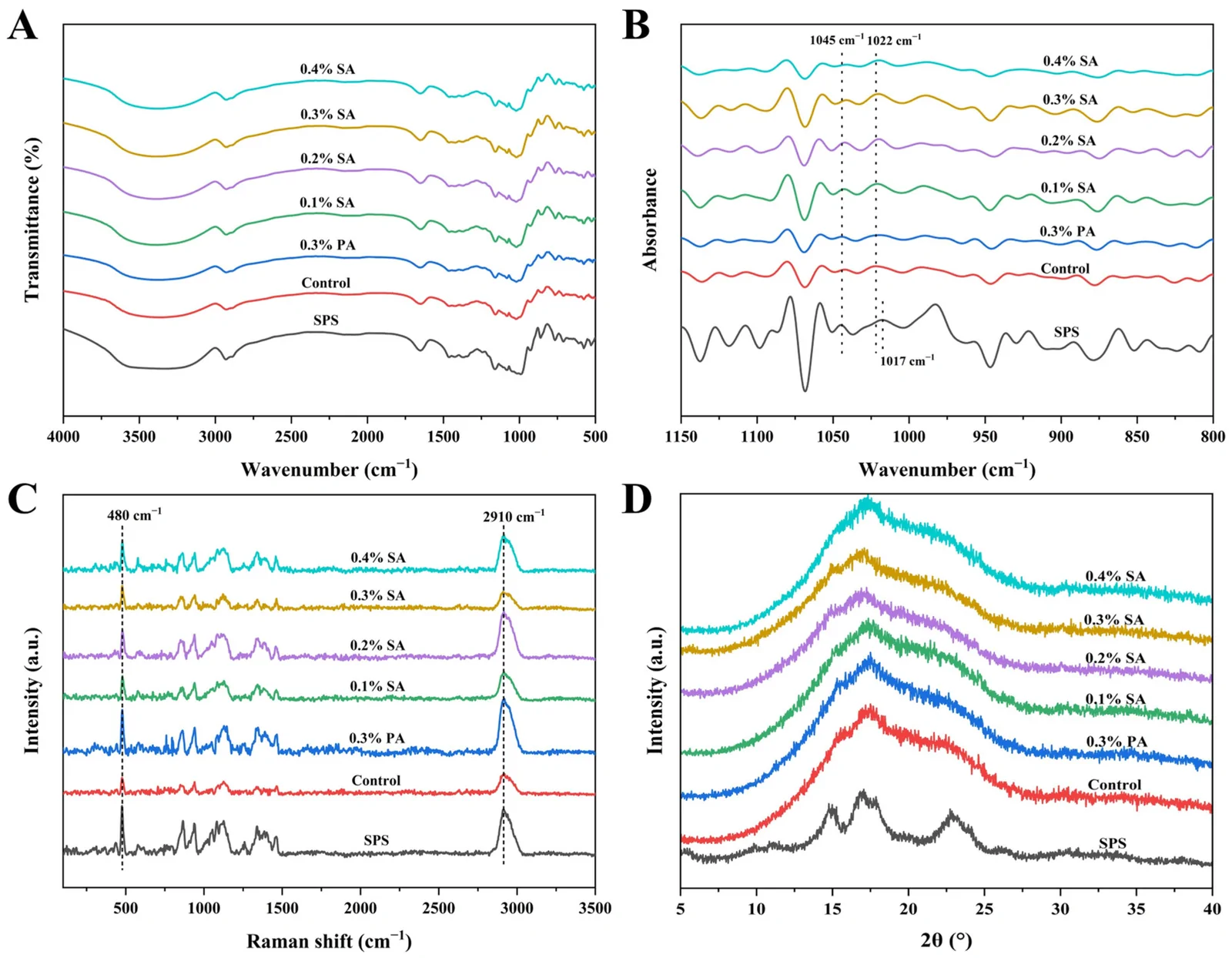
FTIR spectra, Raman spectra, and XRD diffractograms of sweet potato starch (SPS) and wet instant starch noodle cakes (WISNC). (**A**) FTIR spectra; (**B**) deconvoluted FTIR spectra (1150–800 cm^−1^); (**C**) Raman spectra; (**D**) XRD diffractograms. Control: WISNC without additives; 0.3% PA: WISNC with 0.3% potassium alum (*w*/*w*); 0.1–0.4% SA: WISNC containing the corresponding concentrations of sodium alginate (*w*/*w*).

The FTIR spectra of the SPS sample and WISNC samples supplemented with SA and PA are shown in Figure 3A. All samples showed a strong and broad infrared absorption band between 3700 and 3000 cm^−1^, attributed to starch O–H stretching [26]. The O–H peak of SPS was located at 3336.66 cm^−1^, whereas that of the WISNC samples ranged from 3354.22 to 3389.65 cm^−1^. Compared with SPS, the O–H stretching vibration peaks of the WISNC samples shifted to higher wavenumbers, indicating an overall weakening of the intermolecular hydrogen bonding interactions among starch molecules [26,39]. This observation aligns with Yang et al. [26], who also reported a blue shift in the O–H peak in wet sweet potato starch noodles relative to that of SPS during retrogradation. This shift is mainly attributed to the boiling process during noodle forming: the treatment partially disrupted the original intra- and intermolecular hydrogen bond networks of starch, causing the dissociation of ordered molecular structures. Consequently, during the subsequent retrogradation at room and low temperature, the recrystallized structures formed by starch molecular rearrangement exhibited a lower degree of order and reduced stability [40].

To compare the short-range ordered structures of SPS and WISNC samples with different additives, Fourier self-deconvolution was applied to the characteristic spectral bands in the wavenumber range of 1200–800 cm^−1^, and the results are presented in Figure 3B. This absorption region primarily arises from stretching of C–O, C–C, and C–O–C bonds. Due to the high sensitivity of these vibrations to conformational changes and the degree of order in starch polymers, this region is commonly regarded as the fingerprint region for starch-based materials [41]. Qiao et al. [40] and Yang et al. [3] reported that the 1045 cm^−1^ absorption band corresponds to starch’s crystalline region, whereas the 1022/1017 cm^−1^ band reflects its amorphous fraction, indicating that the absorbance ratio (R_1045/1022(1017)_) serves as an indicator of the crystalline structure and short-range order of starch. Table 2 indicates that SPS exhibits a higher R_1045/1017_ than the R_1045/1022_ of all WISNC samples, reflecting decreased starch chain regularity and a broadened bond energy distribution due to gelatinization and recrystallization during processing. Compared with the control WISNC, the 0.3% PA-added group exhibited a significantly higher R_1045/1022_ value (*p* < 0.05), suggesting that PA addition enhanced the short-range order of WISNC. Zhang et al. [22] similarly observed that alum influenced the short-range order in potato starch gel. With increasing SA concentration, the R_1045/1022_ value first increased from 0.664 to 0.732 and then decreased to 0.640, indicating a corresponding change in the short-range order of WISNC. This trend aligns with that reported by Liu et al. [42], who attributed it to the disruption or interference with starch molecular rearrangement and recrystallization by high concentrations of SA.

### 3.5. Raman Analysis

Figure 3C shows the Raman spectra of the SPS sample and WISNC samples supplemented with SA and PA. Distinct characteristic Raman peaks appear at 480, 865, 940, 1264, and 2910 cm^−1^, which can be assigned to the pyranose ring skeletal vibration, C–H deformation vibration, skeletal vibration of C–O–C, C–O and CH_2_OH-related vibrations of the side chain, and C–H stretching vibration, respectively. Among these, the 480 and 2910 cm^−1^ bands are routinely employed to monitor molecular order changes during starch gelatinization and retrogradation [43,44]. Table 2 shows that the SPS peak at ~480 cm^−1^ (474.93 cm^−1^) shifts to higher wavenumbers (477.46–479.52 cm^−1^) in WISNC samples. In contrast, the SPS peak near 2910 cm^−1^ exhibited no consistent trend in peak shifts among the WISNC samples, consistent with the findings of Yang et al. [26], who also observed variable peak-shift behavior in this region for SPS and wet starch noodles stored under different conditions. The observed peak shifts may be associated with structural rearrangements of starch during gelatinization and subsequent retrogradation, which involve not only the disruption of the original helical structure but also the reassembly of molecular chains into a new gel network with a different degree of molecular order, thereby altering the vibrational environment of relevant chemical bonds.

The full width at half maximum (FWHM) serves as a sensitive indicator of structural differences among samples, with band narrowing reflecting higher structural order and a more concentrated bond energy distribution [45]. FWHM_480_ and FWHM_2910_ are widely used to assess starch short-range structural order [44,46]. Table 2 shows that the FWHM_480_ and FWHM_2910_ values of SPS were 16.24 and 96.73, respectively, while those of the control WISNC sample increased to 20.49 and 99.88, respectively. Upon addition of 0.3% PA, these values decreased to 17.86 and 94.51, respectively. The increased FWHM values in the control WISNC suggest a broader and more heterogeneous distribution of local vibrational environments, whereas the addition of PA resulted in narrower Raman bands, suggesting a more homogeneous local molecular environment and greater structural uniformity. This trend is consistent with the increase in the R_1045/1022_ ratio, further supporting the role of PA in promoting starch molecular ordering. Both the FWHM_480_ and FWHM_2910_ reached their minimum values at 0.2% SA, followed by an increasing and then decreasing trend at higher SA concentrations. This trend was inconsistent with the FTIR results and may be attributed to the different structural sensitivities of the two techniques, with Raman FWHM being more sensitive to variations in local vibrational environments, which may be influenced by molecular rearrangement at higher SA concentrations.

### 3.6. XRD Analysis

XRD diffractograms of SPS and WISNC samples appear in Figure 3D. The XRD profile of SPS exhibits a weak diffraction peak at 5.6°, along with strong and sharp diffraction peaks at ~15°, 17°, 18°, and 23°, indicative of C_A_-type crystallinity [26]. Compared with SPS, the XRD pattern of the gelatinized and retrograded WISNC appears more diffuse, with weaker diffraction peaks at ~15°, 17°, 22°, and 24°. This broad diffraction profile suggests that the starch in the noodle cakes is predominantly amorphous, with a relatively low degree of long-range order. This is primarily attributed to the limited ordered packing of double helices formed by amylose and the outer chains of amylopectin during short-term retrogradation, resulting in microcrystalline domains with small size, low crystallinity, and numerous defects. Consistent with these findings, Yang et al. [3] reported that both dried and wet potato starch noodles subjected to gelatinization and retrogradation treatments exhibit significantly reduced diffraction peak intensities and diffuse patterns.

As indicated in Table 3, WISNC samples exhibited much lower crystallinity (2.57–6.10%) compared with SPS (31.92%). Adding 0.3% PA markedly increased WISNC crystallinity relative to the control. This may be ascribed to the hydrolysis of Al^3+^ from PA in aqueous solution, which forms an aluminum hydroxide colloid that adsorbs starch chains, promotes their aggregation and rearrangement, and thus facilitates the long-range order of starch molecules. SA addition also influenced WISNC crystallinity: at 0.1% and 0.3% SA, no significant change was observed relative to the control, whereas 0.2% SA increased crystallinity significantly, and 0.4% SA caused a notable decrease. This indicates that SA modulates retrograded starch crystallinity in a concentration-dependent manner. At an appropriate concentration, SA can act as a physical crosslinker, promoting the orientation and crystallization of starch chains. With increasing SA addition, the crystallinity of WISNC first increased and then decreased, which aligns with the findings of Fu et al. [27]. They reported that the crystallinity of highland barley noodles with 0.6% SA addition was higher than that with 0.3% and 0.9% addition. Li et al. [12] investigated the effect of gelatin on flat potato starch noodles and found that a small amount of gelatin promoted the short-term retrogradation of amylose, whereas excess gelatin led to a decrease in relative crystallinity. They attributed this effect to hydrogen bonding interactions between excess gelatin and amylose, which disrupted amylose interchain associations.

### 3.7. DSC Analysis

The hydrothermal parameters of SPS and WISNC samples are summarized in Table 3. SPS exhibited a solitary endotherm at *T*_o_ 71.5, *T*_p_ 79.0, and *T*_c_ 86.3 °C, corresponding to starch crystallite melting, double-helix unwinding, and hydrogen-bond rupture [5]. For all WISNC samples, an endothermic peak corresponding to the melting of recrystallized amylopectin [47] was detected in the range of 48–64 °C. The *T*_o_ values of the WISNC samples were similar, and their *T*_p_ and *T*_c_ values were generally comparable, although significant differences were observed among some samples. The Δ*H* of SPS was 8.28 J/g, whereas that of WISNC samples was slightly lower, ranging from 5.76 to 7.24 J/g, indicating reduced structural order and thermal stability in the recrystallized starch compared with native starch granules. This aligns with the findings of Yang et al. [26]. Compared with the control group, the addition of 0.3% PA did not significantly affect the ΔH value of WISNC. Regarding WISNC samples with different SA addition levels, the group receiving 0.2% SA exhibited a markedly greater Δ*H* relative to the control, while the Δ*H* values of the 0.3% and 0.4% groups followed a declining pattern, suggesting that the effect of SA on the extent of WISNC retrogradation is concentration-dependent. This conclusion is consistent with the XRD analysis results.

### 3.8. Water Phase Distribution of WISNC Samples

**Figure 4:**
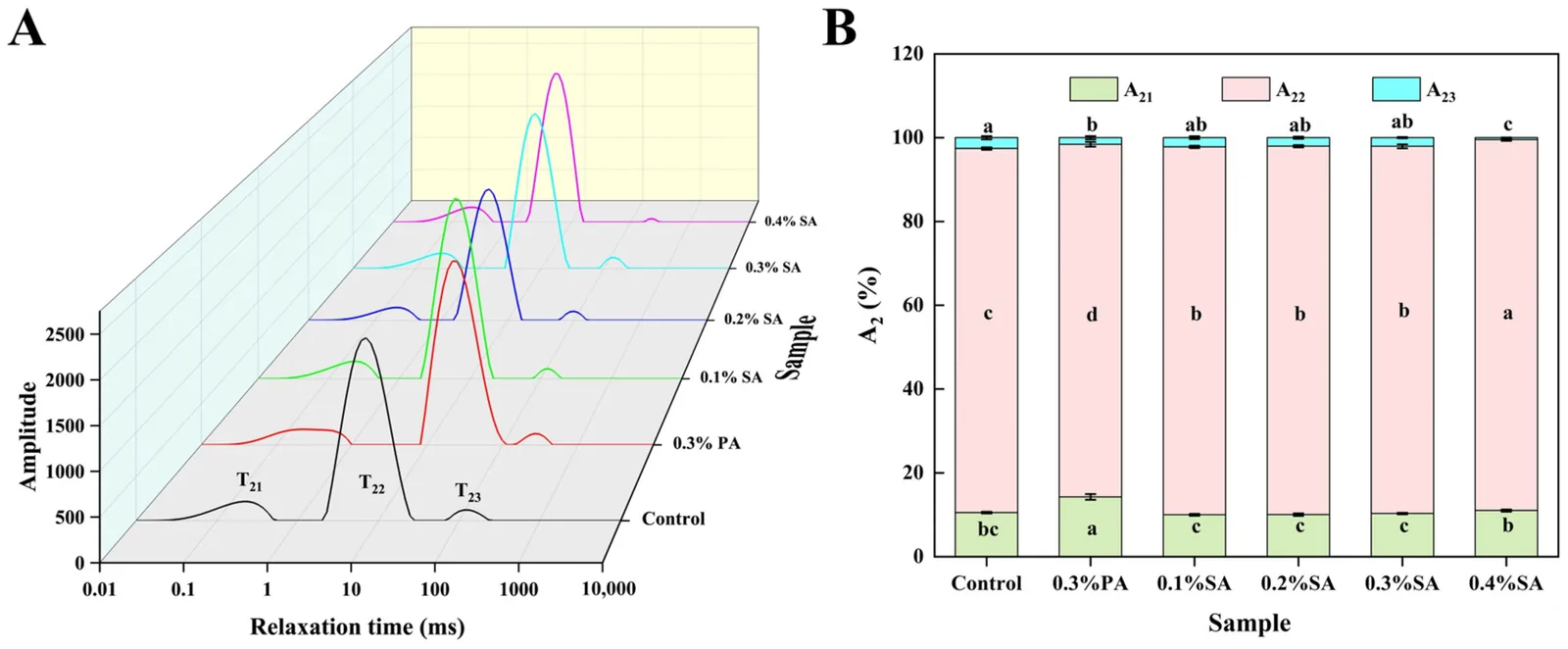
Relaxation time T_2_ (**A**) and peak area ratio A_2_ (**B**) of wet instant starch noodle cakes (WISNC). Different lowercase letters within the same A_2i_ indicate significant differences (*p* < 0.05). Control: WISNC without additives; 0.3% PA: WISNC with 0.3% potassium alum (*w*/*w*); 0.1%, 0.2%, 0.3%, and 0.4% SA: WISNC containing the corresponding concentrations of sodium alginate (*w*/*w*).

T_2_ relaxation time reflects water–matrix interactions, with shorter values denoting stronger water binding. Figure 4 presents the T_2_ relaxation times of WISNC samples with PA and varying SA concentrations, where all samples exhibit three distinct peaks (T_21_, T_22_, and T_23_) in the 0.1–10,000 ms range, corresponding to three different water phases [48,49]. Specifically, T_21_ (0.01–1.52 ms) is attributed to bound water tightly associated with the surfaces of amylopectin and amylose chains; T_22_ (1.74–65.80 ms) corresponds to weakly bound water immobilized by the three-dimensional retrograded gel matrix; and T_23_ (57.22–705.48 ms) is assigned to free water trapped within large pores or defects of the gel network. The relative peak areas, A_21_, A_22_, and A_23_ (%), indicate the relative content of each water phase.

As shown in Figure 4B, A_22_ consistently accounts for the highest proportion (84.18–88.53%) among all WISNC samples, followed by A_21_ (10.00–14.25%) and A_23_ (0.45–2.58%). This distribution is characteristic of typical intermediate water [50], indicating that the water in WISNC is predominantly weakly bound water. This finding aligns with the report by Yang et al. [26], who reported that water with a relaxation time of 8–20 ms dominated in fresh wet starch noodles. Relative to the control group, the PA-added group exhibited a significantly broadened T_21_ peak, accompanied by a higher fraction of bound water and a lower fraction of weakly bound water. This can be ascribed to the interactions between Al^3+^ and starch molecules, together with the hydrogen-bond-mediated adsorption of water molecules onto aluminum hydroxide colloids generated through hydrolysis [7]. These interactions may collectively restrict water mobility within the starch network, altering the distribution of water among different states and favoring a shift in the relative water population from weakly bound toward more tightly bound states. Similarly, Li et al. [35] reported that incorporating PA into potato starch noodles enhanced the proportion of bound water, as reflected by an increased peak area.

The addition of SA significantly increased the fraction of weakly bound water (A_22_) in WISNC samples, suggesting that SA may interact with amylose molecules to alter the gel network microstructure and consequently influence the distribution and mobility of water within the matrix. Moreover, as the SA concentration increased, the free water fraction exhibited a decreasing trend, consistent with the findings of Han et al. [51]. This effect may result from enhanced SA-amylose interactions at higher SA concentrations, which in turn could reduce amylose-amylose interactions, hinder starch retrogradation, and consequently limit the shift in the relative water population from the weakly bound state toward the free-water state [52].

### 3.9. SEM Analysis

**Figure 5:**
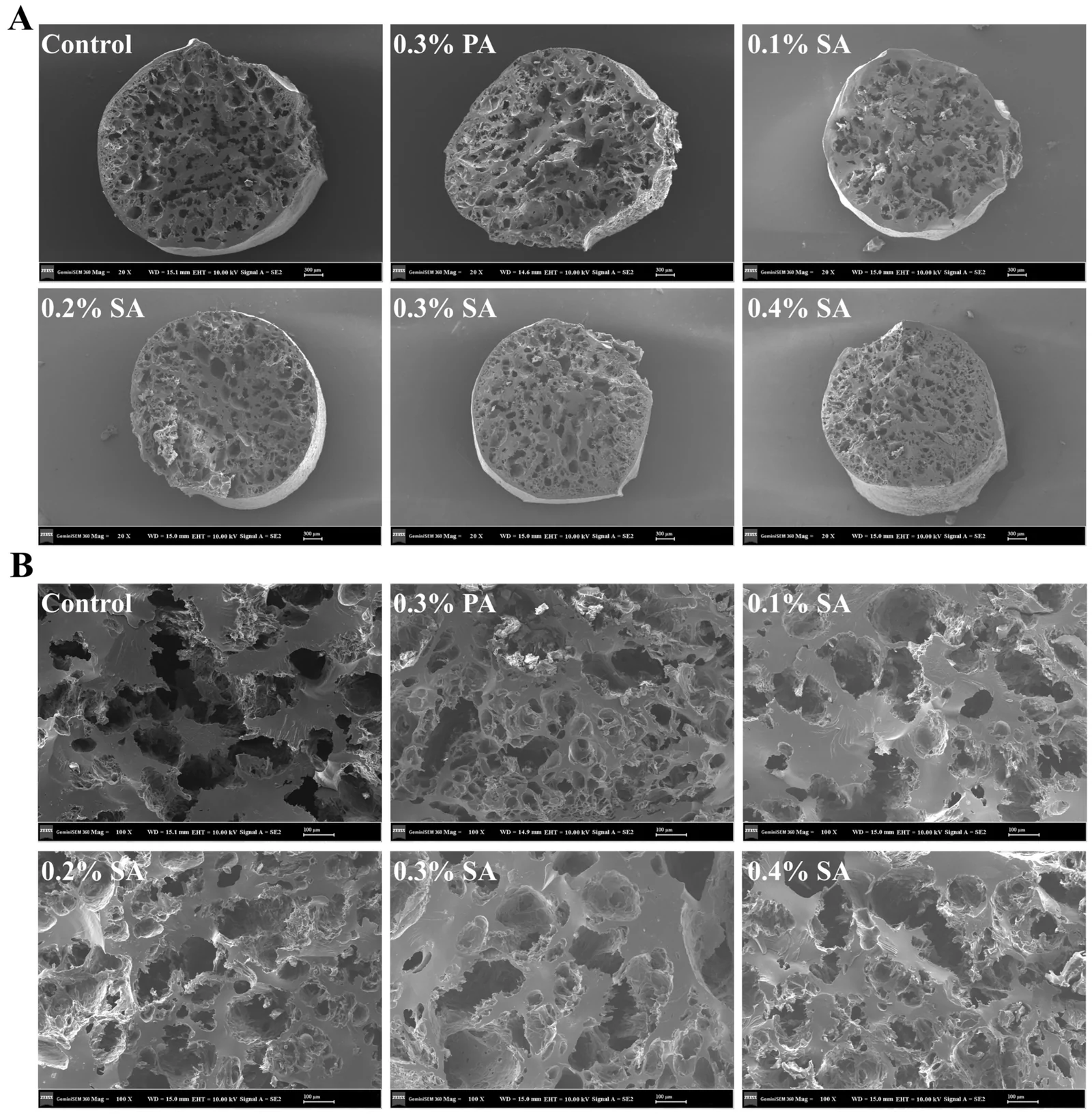
Cross-sectional micrographs of wet instant starch noodle cakes (WISNC): (**A**) 20× magnification; (**B**) 100× magnification. Control: WISNC without additives; 0.3% PA: WISNC with 0.3% potassium alum (*w*/*w*); 0.1%, 0.2%, 0.3%, and 0.4% SA: WISNC containing the corresponding concentrations of sodium alginate (*w*/*w*).

The cross-sectional microstructure of WISNC samples is presented in Figure 5. At 20× magnification (Figure 5A), all samples exhibited irregular pore structures, which resulted from the sublimation of ice crystals formed during the freezing stage. Under higher magnification (Figure 5B), a distinct “honeycomb”-like porous network was observed, indicative of a starch gel network [36]. Similar microstructural features have also been observed in potato starch noodles [53]. The morphology and pore size provide insights into intermolecular interactions [54]. As illustrated in Figure 5B, the 0.3% PA group exhibited a denser network with smaller pores than the control, suggesting that PA effectively alleviated freezing-induced mechanical disruption of the network structure. Addition of 0.1–0.4% SA led to the formation of regular pores. Specifically, 0.2% SA produced relatively small pores, whereas 0.3–0.4% SA resulted in slightly larger pores. These results suggest that an appropriate SA concentration can mitigate the mechanical damage induced by the freezing step on the amylose-based network, whereas excessive SA addition diminishes this protective effect.

### 3.10. Mechanism Underlying the Effect of SA on WISNC Quality

**Figure 6:**
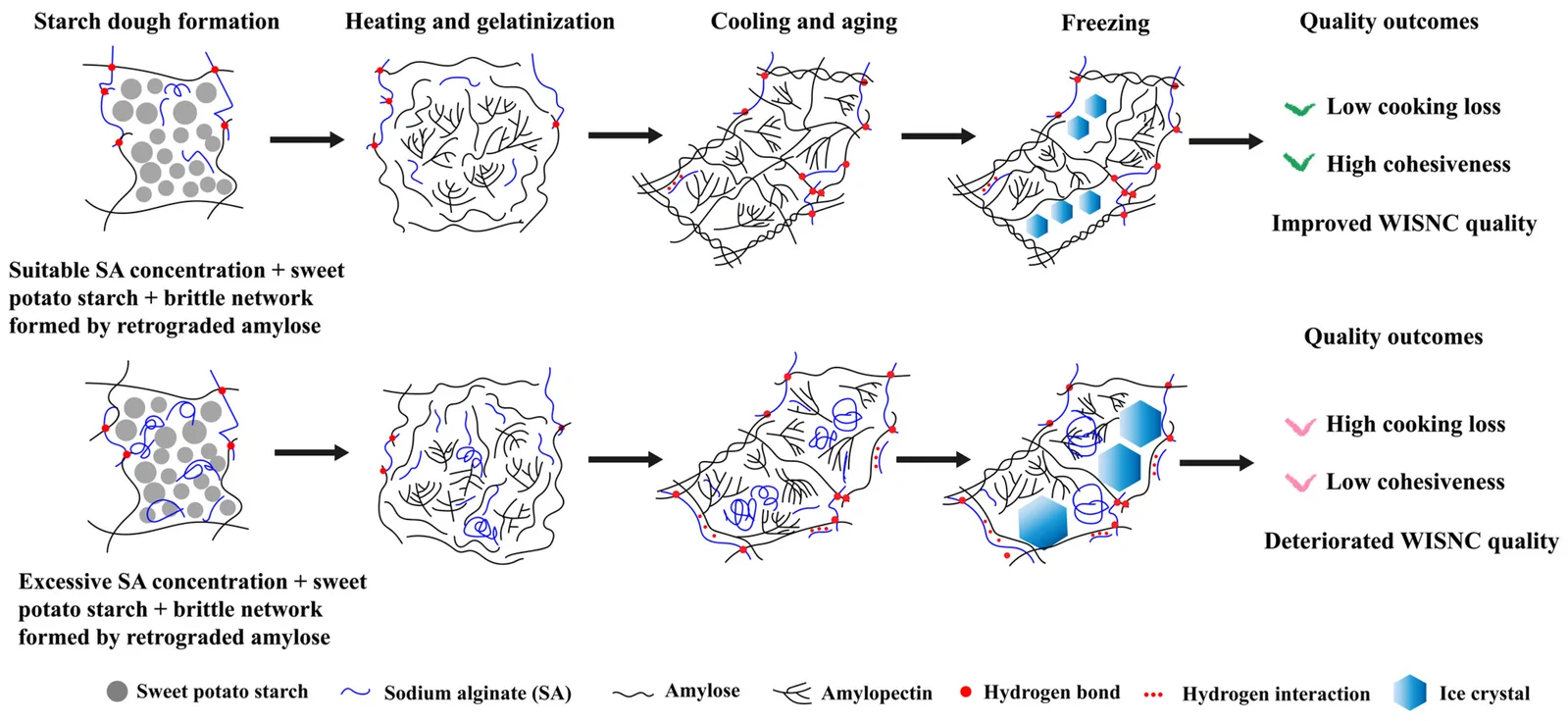
Conceptual model illustrating the potential effects of SA addition on the structural evolution and quality attributes of WISNC.

Figure 6 presents a proposed conceptual model illustrating how SA addition influences the quality attributes of WISNC, based on the rheological changes in the starch dough and multi-scale structural changes in WISNC. The addition of an appropriate concentration (0.2%) of SA may enhance intermolecular interactions within the starch–SA system during starch dough preparation, favoring the formation of a more supportive molecular network and potentially improving dough strength. This interpretation is supported by the frequency- sweep rheological results, which showed an increase in the n value (Table 1). After the starch dough is dropped into boiling water, the starch granules begin to absorb water and swell, while amylose leaches out from the starch granules. Upon cooling and aging of the starch noodles, an appropriate concentration of SA may facilitate the aggregation and oriented alignment of amylose molecules, contributing to the formation of a more uniform and dense gel network. During subsequent freezing, ice crystal formation in the WISNC drives structural evolution. Free water crystallizes preferentially, excluding starch and SA into the unfrozen phase, leading to freeze concentration and water redistribution. The preformed SA–starch gel network may restrict water mobility during freezing, potentially reducing freeze-induced structural damage and modifying the freeze-induced pore structure. This may help preserve the structural integrity of the amylose-based gel network. SEM micrographs provide structural evidence for this interpretation, showing a relatively dense network structure with smaller voids (Figure 5). This structural preservation was conducive to enhanced short-range and long-range structural ordering, as indicated by increased R_1045/1022_, relative crystallinity, and ΔH (Table 2 and Table 3). These structural changes were accompanied by reduced cooking loss and increased cohesiveness of WISNC.

However, excess SA may compete with starch molecules for available water, increasing hydration heterogeneity within the starch dough and potentially reducing its structural stability, consistent with the increase in the m value (Table 1). During the cooling and aging of the starch noodle cakes, excess SA may further interfere with the approach, alignment, and reorganization of amylose molecules, leading to the formation of a less ordered and less dense gel network. Upon entering the freezing stage, the weakened gel network may have a reduced capacity to immobilize water and constrain ice crystal growth, which could be associated with greater structural disruption of the starch matrix. This structural disruption was accompanied by limited development of ordered short-range and long-range structures, as reflected by reduced R_1045/1022_, relative crystallinity, and ΔH (Table 2 and Table 3), together with increased cooking loss and reduced cohesiveness of WISNC.

### 3.11. Aluminum Residue Analysis

The aluminum residues in SPS and WISNC samples are shown in Figure S2. The aluminum content of the original SPS was 31.18 mg/kg, slightly exceeding the range of 2.42–30.00 mg/kg reported by Mu et al. [55], who demonstrated that the aluminum content of SPS varied considerably depending on the starch isolation process. Following processing into Control WISNC, the residual aluminum content increased to 48.44 mg/kg. This increase may be partly attributable to aluminum introduced from the dropping tool used during noodle processing. The addition of 0.3% PA markedly increased the aluminum content of WISNC to 212.72 mg/kg, exceeding the maximum limit of 200 mg/kg specified for starch noodles in GB 2760–2024. These results highlight the importance of considering both the intrinsic aluminum content of starch and potential aluminum contamination during processing. Notably, the aluminum residue of the 0.2% SA group was 62.03 mg/kg, which was significantly higher than those of the other SA groups (41.28–44.02 mg/kg) and the control group (48.44 mg/kg). This relatively high aluminum residue may be related to the distinct rheological and structural characteristics induced by 0.2% SA, which could have promoted aluminum migration or retention during processing. Although the aluminum content of the 0.2% SA group was higher than those of the other SA groups, it remained well below the maximum limit of 200 mg/kg specified in GB 2760–2024. Therefore, the above results demonstrate that replacing the added 0.3% PA with 0.2% SA substantially reduced the aluminum content of WISNC while maintaining favorable quality attributes.

## 4. Conclusions

This study systematically evaluated the feasibility of substituting PA with SA by comparing their effects on starch dough rheology, as well as on the rehydration time, cooking quality, textural properties, and multi-scale structure of WISNC. The results demonstrated that PA enhanced starch short-range order and crystallinity, forming a denser gel network with an increased proportion of bound water. These changes reduced the cooking loss of WISNC but markedly prolonged the rehydration time. In contrast, SA exhibited a concentration-dependent effect. Notably, 0.2% SA enhanced starch structural ordering and gel network integrity, resulting in lower cooking loss, increased TPA cohesiveness, and significantly faster rehydration of WISNC. However, excessive SA levels reduced structural order and weakened the gel network, leading to increased cooking loss and reduced TPA cohesiveness. Compared with WISNC samples supplemented with 0.3% SA, the addition of 0.2% SA shortened the rehydration time while maintaining comparable cooking loss and TPA cohesiveness, and reduced the potential health risks associated with aluminum addition. Therefore, 0.2% SA could serve as a potential alternative to added PA for improving the quality of WISNC. However, the use of a single PA concentration (0.3%) limits the interpretation of the comparison between SA and PA, as the optimal PA concentration for WISNC production remains unclear. Furthermore, considering the potential interactions between SA and amylose and their possible implications for starch digestibility, future studies will further investigate the effects of SA addition on the digestive properties of WISNC.

## Figures and Tables

**Table 1:** Linear viscoelastic region (LVER) and power-law model fitting parameters of starch doughs supplemented with PA and different concentrations of SA.

Sample	LVER (% Strain)	*m*	*n*	*R* ^2^
Control	0.028 ± 0.002 ^c^	0.085 ± 0.003 ^d^	6.216 ± 0.015 ^c^	0.972 ± 0.003
0.3% PA	0.047 ± 0.006 ^b^	0.116 ± 0.006 ^b^	6.325 ± 0.028 ^a^	0.952 ± 0.018
0.1% SA	0.028 ± 0.008 ^c^	0.107 ± 0.002 ^c^	6.227 ± 0.024 ^bc^	0.943 ± 0.010
0.2% SA	0.068 ± 0.006 ^a^	0.088 ± 0.004 ^d^	6.282 ± 0.021 ^ab^	0.924 ± 0.006
0.3% SA	0.018 ± 0.002 ^c^	0.123 ± 0.004 ^ab^	6.209 ± 0.029 ^c^	0.946 ± 0.009
0.4% SA	0.021 ± 0.002 ^c^	0.126 ± 0.002 ^a^	6.180 ± 0.030 ^c^	0.956 ± 0.005

Letters that differ within a column denote a statistically significant difference at *p* < 0.05; *m* and *n* are constant values; *R*^2^ represents the coefficient of determination. Control: starch dough without additives; 0.3% PA: starch dough containing 0.3% potassium alum (*w*/*w*); 0.1–0.4% SA: starch dough containing the corresponding concentrations of sodium alginate (*w*/*w*).

**Table 2:** FTIR deconvolution, Raman shift and full width at half maximum (FWHM) of sweet potato starch (SPS) and wet instant starch noodle cakes (WISNC).

Sample	R_1045/1022(1017)_	Raman			
		Shift_480_	FWHM_480_	Shift_2910_	FWHM_2910_
SPS	0.806 ± 0.010 ^a^	474.93 ± 0.24 ^b^	16.24 ± 0.41 ^e^	2914.18 ± 1.07 ^bc^	96.73 ± 0.70 ^c^
Control	0.664 ± 0.025 ^d^	477.46 ± 1.40 ^a^	20.49 ± 0.46 ^a^	2918.91 ± 0.59 ^a^	99.88 ± 0.56 ^b^
0.3% PA	0.780 ± 0.010 ^b^	478.33 ± 0.71 ^a^	17.86 ± 0.57 ^cde^	2918.53 ± 0.23 ^a^	94.51 ± 0.64 ^d^
0.1% SA	0.718 ± 0.013 ^c^	479.38 ± 0.44 ^a^	20.09 ± 0.11 ^ab^	2914.30 ± 1.24 ^bc^	103.03 ± 0.48 ^a^
0.2% SA	0.732 ± 0.005 ^c^	479.52 ± 0.58 ^a^	17.17 ± 0.06 ^de^	2914.89 ± 0.40 ^bc^	98.01 ± 0.53 ^bc^
0.3% SA	0.667 ± 0.009 ^d^	478.26 ± 1.03 ^a^	19.21 ± 0.62 ^abc^	2912.05 ± 1.65 ^c^	99.94 ± 0.21 ^b^
0.4% SA	0.640 ± 0.013 ^e^	479.31 ± 1.05 ^a^	18.40 ± 0.65 ^bcd^	2916.86 ± 1.87 ^ab^	99.24 ± 0.34 ^b^

Different letters in the same column indicate significant differences (*p* < 0.05). Control: WISNC without additives; 0.3% PA: WISNC with 0.3% potassium alum (*w*/*w*); 0.1%, 0.2%, 0.3%, and 0.4% SA: WISNC containing the corresponding concentrations of sodium alginate (*w*/*w*).

**Table 3:** Crystallinity and thermal properties of sweet potato starch (SPS) and wet instant starch noodle cakes (WISNC).

Sample	Crystallinity (%)	DSC			
		*T*_o_ (°C)	*T*_p_ (°C)	*T*_c_ (°C)	Δ*H* (J/g)
SPS	31.92 ± 0.81 ^a^	71.5 ± 0.8 ^a^	79.0 ± 0.2 ^a^	86.3 ± 0.9 ^a^	8.28 ± 0.31 ^a^
Control	4.31 ± 0.05 ^de^	48.4 ± 0.6 ^b^	55.3 ± 0.2 ^bc^	61.7 ± 0.1 ^bc^	6.64 ± 0.10 ^cd^
0.3% PA	5.30 ± 0.07 ^c^	48.3 ± 0.1 ^b^	55.8 ± 0.4 ^b^	63.5 ± 1.1 ^b^	6.90 ± 0.20 ^bc^
0.1% SA	4.71 ± 0.04 ^cd^	48.5 ± 0.0 ^b^	55.0 ± 0.1 ^c^	62.5 ± 0.2 ^bc^	5.76 ± 0.16 ^e^
0.2% SA	6.10 ± 0.15 ^b^	48.3 ± 0.8 ^b^	55.6 ± 0.3 ^b^	61.6 ± 1.1 ^c^	7.24 ± 0.07 ^b^
0.3% SA	3.80 ± 0.26 ^e^	48.7 ± 0.4 ^b^	55.4 ± 0.1 ^bc^	62.1 ± 0.6 ^bc^	6.31 ± 0.23 ^d^
0.4% SA	2.57 ± 0.20 ^f^	49.2 ± 0.6 ^b^	55.3 ± 0.0 ^bc^	62.4 ± 0.2 ^bc^	6.20 ± 0.29 ^de^

Data in the same column with different superscript letters indicate a statistically significant difference (*p* < 0.05). *T*_o_: onset temperature; *T*_p_: peak temperature; *T*_c_: conclusion temperature; Δ*H*: enthalpy change. Control: WISNC without additives; 0.3% PA: WISNC with 0.3% potassium alum (*w*/*w*); 0.1%, 0.2%, 0.3%, and 0.4% SA: WISNC containing the corresponding concentrations of sodium alginate.

## Data Availability

The original contributions presented in this study are included in the article/Supplementary Materials. Further inquiries can be directed to the corresponding authors.

## Supplementary Materials

The following supporting information can be downloaded at: https://www.mdpi.com/article/10.3390/foods15193490/s1, Figure S1: Process flow diagram of wet instant starch noodle cakes (WISNC); Figure S2: Aluminum residues in sweet potato starch (SPS) and wet instant starch noodle cakes (WISNC). Control: WISNC without additives; 0.3% PA: WISNC with 0.3% potassium alum (w/w); 0.1%, 0.2%, 0.3%, and 0.4% SA: WISNC containing the corresponding concentrations of sodium alginate (w/w).
