# Supplementary material for: Effects of Sodium Alginate on the Quality Attributes of Wet Instant Starch Noodle Cakes: From a Multi-Scale Perspective
Source: Foods. 2026 Sep 29;15(19):3490. doi: 10.3390/foods15193490 (PMC13664315; doi:10.3390/foods15193490)
Supplement: Supplementary file 1 [file foods-15-03490-s001.zip › foods-4526505-supplementary.pdf]

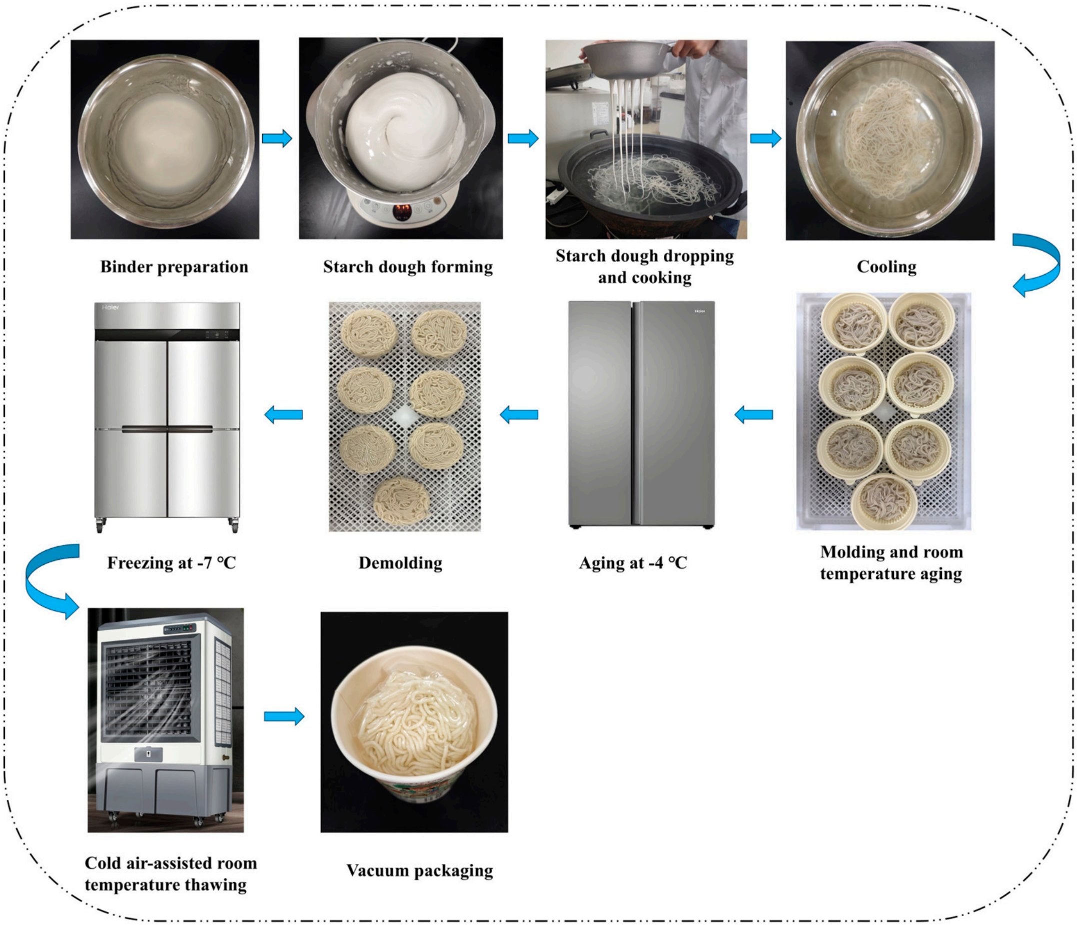

Figure S1. Process flow diagram of wet instant starch noodle cakes (WISNC).

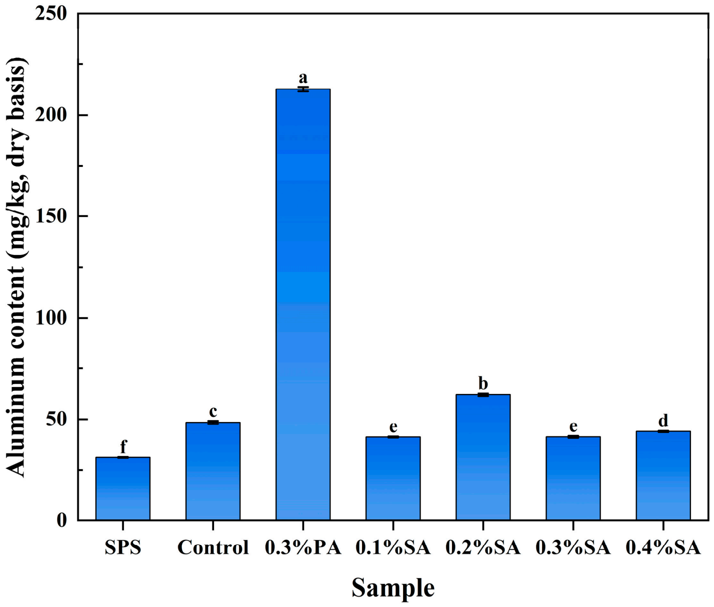

Figure S2. Aluminum residues in sweet potato starch (SPS) and wet instant starch noodle cakes (WISNC). Control: WISNC without additives; 0.3% PA: WISNC with 0.3% potassium alum (w/w); 0.1%, 0.2%, 0.3%, and 0.4% SA: WISNC containing the corresponding concentrations of sodium alginate (w/w).
